# INFLUENCE OF PHYSICAL THERAPY REHABILITATION ON FUNCTIONAL RECOVERY OF OLDER PEOPLE AFTER COVID-19

**DOI:** 10.1093/geroni/igad104.3433

**Published:** 2023-12-21

**Authors:** Gabriela Ochiai, Erika Gouveia E Silva, Daniella De Melo, Diêgo Araújo Ramos, Fabio Cavalcanti Freitas, Vivian Cintra Sousa, Ana Carolina Schmitt, Jose Pompeu

**Affiliations:** University of Sao Paulo, Sao Paulo, Sao Paulo, Brazil; University of Sao Paulo, Sao Paulo, Sao Paulo, Brazil; University of Sao Paulo, Sao Paulo, Sao Paulo, Brazil; University of Sao Paulo, Sao Paulo, Sao Paulo, Brazil; University of Sao Paulo, Sao Paulo, Sao Paulo, Brazil; University of Sao Paulo, Sao Paulo, Sao Paulo, Brazil; University of Sao Paulo, Sao Paulo, Sao Paulo, Brazil; University of Sao Paulo, Sao Paulo, Sao Paulo, Brazil

## Abstract

Retrospective cohort conducted through an online survey sent to older people (≥60 years) of both sexes, with confirmed diagnosis of Covid-19 registered in the Municipal Health Department of Sao Paulo, Brazil. Individuals with cognitive limitations and those who refused to participate were excluded. Functional capacity was assessed using Katz and Lawton Indices for pre-Covid, immediate post-Covid, and current time points, and characteristics of post-Covid physiotherapeutic rehabilitation were collected. Statistical analysis was performed using JASP-0.17.3 software, and group comparisons were made using repeated measures ANOVA, considering alpha=0.05. From March to August 2023, 86 individuals participated, with median age of 66.5 [62.3-71.0] and 57.0% (n=49) male. Among them, 39.5% (n=34) received recommendations for physiotherapeutic rehabilitation and 88.2% (n=30) of them were rehabilitated, while 11.8% (n=4) did not receive treatment. Older people with recommendation for physiotherapy post-Covid-19 demonstrated worse functional status (p< 0.001). Older people who received treatment were functionally equivalent to those who did not attend physiotherapy rehabilitation (p=1.000 for Katz Index and p=0.812 for Lawton Index). The primary indication for post-Covid-19 physiotherapy was muscular fatigue (70.0%, n=21), and domiciliary care was the predominant modality received (46.7%, n=14). Older people who were recommended for physiotherapy showed worse functional status after Covid-19, but there was no difference in long-term functional capacity. This suggests the potential importance of physiotherapeutic rehabilitation for functional recovery after Covid-19.

